# A Weather Forecast Model Accuracy Analysis and ECMWF Enhancement Proposal by Neural Network

**DOI:** 10.3390/s19235144

**Published:** 2019-11-24

**Authors:** Jaroslav Frnda, Marek Durica, Jan Nedoma, Stanislav Zabka, Radek Martinek, Michal Kostelansky

**Affiliations:** 1 Department of Quantitative Methods and Economic Informatics, Faculty of Operation and Economics of Transport and Communications, University of Zilina, 01026 Zilina, Slovakia; marek.durica@fpedas.uniza.sk; 2 Department of Telecommunications, Faculty of Electrical Engineering and Computer Science, VSB –Technical University of Ostrava, 70833 Ostrava-Poruba, Czech Republic; stanislav.zabka@vsb.cz (S.Z.); michal.kostelansky.st@vsb.cz (M.K.); 3 Department of Cybernetics and Biomedical Engineering, Faculty of Electrical Engineering and Computer Science, VSB—Technical University of Ostrava, 70833 Ostrava-Poruba, Czech Republic; radek.martinek@vsb.cz

**Keywords:** ALADIN, ECMWF, neural networks, weather forecast models

## Abstract

This paper presents a neural network approach for weather forecast improvement. Predicted parameters, such as air temperature or precipitation, play a crucial role not only in the transportation sector but they also influence people’s everyday activities. Numerical weather models require real measured data for the correct forecast run. This data is obtained from automatic weather stations by intelligent sensors. Sensor data collection and its processing is a necessity for finding the optimal weather conditions estimation. The European Centre for Medium-Range Weather Forecasts (ECMWF) model serves as the main base for medium-range predictions among the European countries. This model is capable of providing forecast up to 10 days with horizontal resolution of 9 km. Although ECMWF is currently the global weather system with the highest horizontal resolution, this resolution is still two times worse than the one offered by limited area (regional) numeric models (e.g., ALADIN that is used in many European and north African countries). They use global forecasting model and sensor-based weather monitoring network as the input parameters (global atmospheric situation at regional model geographic boundaries, description of atmospheric condition in numerical form), and because the analysed area is much smaller (typically one country), computing power allows them to use even higher resolution for key meteorological parameters prediction. However, the forecast data obtained from regional models are available only for a specific country, and end-users cannot find them all in one place. Furthermore, not all members provide open access to these data. Since the ECMWF model is commercial, several web services offer it free of charge. Additionally, because this model delivers forecast prediction for the whole of Europe (and for the whole world, too), this attitude is more user-friendly and attractive for potential customers. Therefore, the proposed novel hybrid method based on machine learning is capable of increasing ECMWF forecast outputs accuracy to the same level as limited area models provide, and it can deliver a more accurate forecast in real-time.

## 1. Introduction 

### Background and Motivation 

Climate change and global warming have been becoming global issues since the last decade. Weather modelling and prediction are essential especially for aviation services, region/city crisis management, or agricultural sector. Forecasting services may soon start adding information about the effects of climate change to their outputs. The aim is to provide information about how extreme weather events relate to climate change.

Numerical weather prediction (NWP) model has been designated to estimate the future atmospheric behaviour based on the current state and mathematical and physics principles by using data collected from meteorological and aerological stations. The investigated area is transformed into a grid with horizontal resolution (geographical distance between two predicted points) shifting from 10 km for global models to less than 5 km for regional models. The resolution of one grid “cell“ indicates how precisely the terrain can be approximated by the model. Each cell gets its own set of computed parameters, namely temperature, precipitations, humidity, pressure, as well as wind speed and direction. Based on the grid resolution size and forecast period, models can be divided as follows:

**Global weather forecast models**—resolution of 9 × 9 km and more, forecast period 10–15 days (long-range models provide weather information for a period of more than one month).

**Regional weather forecast models**—standard 5 × 5 km resolution, provide up to three days forecast.

NWP model must calculate on a three-dimensional grid of points. The closer the points are to each other, the better the model characterises the atmosphere and topography. However, small horizontal grids need more vertical levels. This requires more computing power.

**Figure 1:**
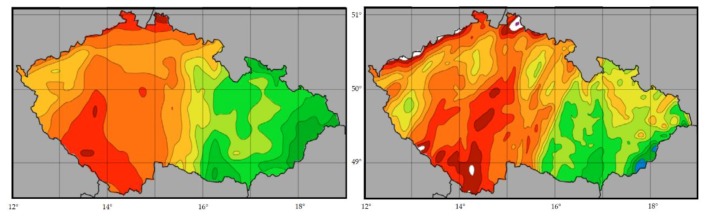
Comparison of the European Centre for Medium-Range Weather Forecast (ECMWF) (left) and ALADIN (right) precipitation forecast. Dark colours represent a large amount of daily precipitation.

Prediction within a very short time period (up to 12 h) is known as nowcasting prediction. This type of forecast models can have a very high-resolution of 1 x 1 km, helps to reduce potential costs and losses in economic activities. The disadvantage of such a resolution type lies in a significant raise of required computer performance, hence nowcasting cannot be solved by the numeric forecast model for a longer period. Figure 1 shows the forecast of precipitation distribution for the Czech Republic. ALADIN model (right) provides more detailed precipitation forecast than ECMWF (left) [1].

Global weather forecast models include ECMWF (UK), GFS (Global Forecast System, USA), or RHMC (Hydrometeorological Center of Russia). In 2016, the ECMWF group released an upgraded system with almost doubled resolution (from 16 to 9 km). Other models work with lower resolution from 13 km (GFS) to 250 km (RHMC) [1,2]. 

Especially in Europe, several regional models are presented, such as ALADIN (founded in 1990 in France), Skiron (University of Athens), or HIRLAM (High-resolution limited area model, Nordic European countries). One of them—ALADIN—is operated by 16 European and North African national meteorological services nowadays. The most important advantages of this local forecast model are [1,3]:

- The region of interest (model domain) can be freely defined, as well as the range and frequency of forecasts (ALADIN provides updates four times per day, ECMWF two times per day);
- the spatial and temporal resolution of forecasts is freely definable (according to the available computing power);
- a focus on regional physical processes is enabled (ECMWF is used for lateral boundary conditions);
- possibility of local observations usage, e.g., radar and satellite for more accurate prediction.

Each of the member countries makes a prediction individually to meet its own criteria. No common application/space containing output predicted data presentation has been created so far, which results in ineffective forecast search on each country’s national meteorological service website separately [3]. On the other hand, there are several accessible applications that use global systems and allow checking of the weather forecast for any place (windy.com, weather.com, yr.no, wunderground.com, etc.). This is the reason why we have decided to prepare the enhancement of global model ECMWF (it has better historical performance than GFS and serves as one of ALADIN limited geographical model inputs). Our aim was to rise up the prediction accuracy by using additional input parameters for better representation of the area belonging to a model grid cell. This will lead to usage of easily accessible global numeric model capable of making forecast for more than one country with accuracy close to regional numerical models.

**Figure 2:**
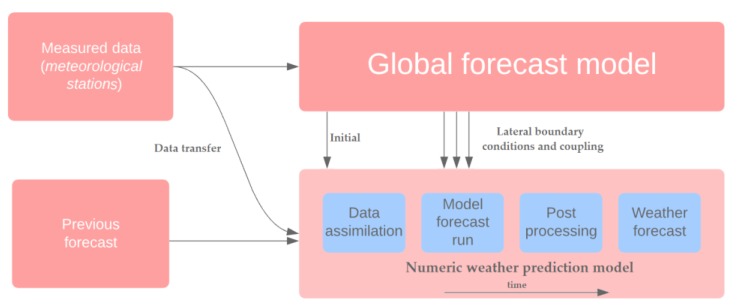
Forecast run scheme of local numeric model.

The ECMWF institution was established in 1975 in the United Kingdom. It computes and disseminates numerical weather predictions to its 34 member states. This data is fully accessible to the national meteorological services operating in the member states [1]. General forecast run scheme of local numerical weather model is depicted by Figure 2.

On the resolution size, we see major weakness of the ECMWF model. The Central Europe region (Slovakia, Poland, Czech Republic, Hungary, Austria, Germany, Switzerland, and Slovenia) has 164 million inhabitants—but only 45 cities with a population over 250,000 (in Slovakia, Hungary, Austria, Switzerland, and Slovenia only their capital cities are more populated) [4]. In this case, one or two grid points (one grid cell represents 81 square km) describe the whole small or medium-size town, which inadequately approximates the terrain and local phenomenon (leeward and windward side of a mountain, green and concrete areas). Every grid point is a combination of horizontal resolution (distance between two horizontal points) and vertical resolution (division of the atmosphere into layers in terms of pressure). 

Our first step for the creation of an enhanced hybrid algorithm consists of data collection. We used the data obtained from digital sensors of a second-generation automatic weather station (AWS) operated by Slovak and Czech hydrometeorological institute. These devices work by using intelligent sensors to measure atmospheric conditions such as air temperature, precipitation, atmospheric pressure, and even wind speed and direction. The second generation of AWS is based on datalogger that measures the sensors, collects and stores data, and controls peripherals. Sensors have a wide operating temperature range, contain wind vector, wet bulb, and rain gage. Histogram is a standard data visualization output and datalogger that allows to set the execution intervals and ample input for each of the selected sensors. The automated system is able to run unnoticed for weeks and months, recording all details of the weather continuously. Data are typically stored in the preferred units (e.g., wind speed in km/s, temperature in °C, or precipitation in mm/h). Data recording intervals are programmable, e.g., hourly and daily data values. Detailed overview of the used sensors is available in [5].

The created database required data mining and data processing techniques such as data cleaning or clustering (based on intra-class similarity). Clear data was visualized, which helped us to extract interesting knowledge from the data in a large database (e.g., potential relations between measured parameters, constraints). We provided data reduction, selecting air temperature and precipitations as useful features. All this information helps to classify the gap between weather prediction and real measured data, and points to trend analysis of prediction error making. Elimination of this phenomenon is based on neural network classification model [6].

The final database was a combination of advanced signal and data processing. During the initialization phase of developing the algorithm, we continued with sensor data gathering for another weather station, which served us for cross-validation and prediction accuracy verification.

This way of data sensors processing is actual and used in many research fields. Outputs gained from these automated weather sensors lead to develop the neural network model. 

Parameters of the selected sensors, along with standard ECMWF output data, helped us to design a multiple layer perceptron neural network, which seemed to be an effective solution (in a form of computing time and power needs, as well as high scalability) for better adaptation of the global prediction model in local conditions, especially in urban areas [6,7].

Replacing the main advantage of a regional model (better scalability of the limited area) with multilayer neural network (hereinafter referred to as NN) model is a new research issue. Recent development of NN algorithms and upgrades of computer hardware led to the study of the wide spectrum of NN deployment [7]. Neural network model can fit general distributions while retaining local details. This paper presents a real-time neural network architecture to predict meteorological parameters based on the latest ECMWF data collection and additional parameters described in Section 3. This NN model can self-train and adapt to changes in the urban environment or air quality that influences the local weather significantly. Not all member countries’ national weather services provide free access to ALADIN model and of course, all members must pay extra for the computing power (and equipment) necessary for ALADIN forecast run. On the other hand, ECMWF numerical forecast outputs are freely accessible e.g., by website *windy.com.* Therefore, the main objective of the proposed model is development of real-time enhancement for ECMWF prediction (mainly for urban areas), providing accuracy similar to regional model ALADIN.

This paper is structured as follows: The *State of the art* section presents published results related to this research topic, typically weather numeric model comparison or analysis of the relation between air quality, weather conditions, and human health. *Methodology* section describes dataset creation along with design and evaluation of the proposed neural network. *Results* section shows an accuracy comparison of our system with a forecast obtained from global and regional weather models. *Discussion* and *Conclusion* sections summarize the proposed model efficiency and future ideas related to this topic.

## 2. State of the Art

Current research activities in this field principally deal with two investigation areas. The first area of research is focused on forecast accuracy obtained from global or regional weather numeric models. There are several studies describing the numeric model’s evaluation results in case of convective precipitations, heavy rainfall and thunderstorms, or strong wind. This information is important for rescue services, forest or mountains visitors, or transportation services. D. Saur [2] evaluated the forecast accuracy for global systems and regional ALADIN system for the Czech Republic and Slovakia. As for our model evaluation purposes, we used several published methods. In paper [8], all participating members of ALADIN surveyed the output data from the time period 2015–2016, and pointed to better accuracy and less RMSE (root mean square error) value in comparison to the global ECMWF model. Piotr Sekula et al. in [9] compared the data gained from meteorological stations (in 2018) and ALADIN outputs, which led to a significant advance in terms of air temperature forecast in High Tatras area. Such a progress was achieved thanks to higher horizontal resolution and better adaptability on altitude caused by applying more vertical levels. Research paper [10] deals with the usage of regional models as a part of an early warning system for flooding forecast and hydrological modelling. Study [11] published critical incident techniques aimed to identify and manage potential weather warnings. Authors identified several critical incidents ranging from tornado, winter storm to flooding, and heat temperature. They implemented a tool containing additional information to provide a reasonable decision for emergency managers who rely primarily on outputs from weather models. 

Air pollution affects the life of the whole society. It has significant impact on health and economic development of a country. Air pollution is made up from mixture of gases and particles in harmful amounts that are released into the atmosphere due to either natural or human activities. High concentration of greenhouse gases leads to global warming, which causes the rise of sea level and extreme weather conditions. Air pollution control and improving of air quality has become the concern of scientists globally nowadays. There are several studies focusing on the investigation of urban air pollution influence on temperature and precipitation. 

Research work [12] shows how weather changes influence air quality in the USA for a long-term measurement period. The paper proves that particulate air pollution increases deaths needlessly. Papers [13] and [14] quantify the impact of air quality (PM_2.5_, ozone) on urban areas. According to the authors, ozone and nitrogen dioxide increase when daily temperature reaches over 20 °C, and particulate matter has a negative effect on precipitation. The issue of heat generation in specific small size city zones known as urban heat island (UHI—often specified as the air temperature dissimilarity between the city and the paralleling air temperature of its surroundings) is discussed in [15]. 

This work investigated the relationship between measured air temperature and the surrounding environment in cities (water, road, building, and vegetation area). Regional climate and local characteristics of urban development can affect the quality of living [16]. Authors prepared a model for temperature or air humidity prediction in specific city areas with regards to the soil profile and day phase (sunset, sunlight, noon) for Spanish city Bilbao. The potential of vegetation and green urban areas remains the most effective approach for air temperature raising mitigation. J.F Bastin et al. [17] assume that massive planting of trees can store million tons of greenhouse gasses and slow down the increase of global air temperature. The New Urban Agenda published by the United Nations [18] describes urban green infrastructure as a major instrument towards a sustainable city climate. Vegetation enhances the environmental conditions of cities by reducing ambient temperatures and improving the overland water flow. In research article [19] authors created a prediction model for the climate of major cities in 2050. They found out that majority of selected cities around the world would shift towards the warmer climate, and that cities lying in the south part of Europe would reach sub-tropical climate without additional actions in urban planning. Work [20] quantifies the contribution of huge traffic and PM particles to air quality, including also analysis of the pollution sources together with the ways to manage and reduce them in inner-city zones.

We were not the first ones to ask a question if machine learning techniques could substitute forecast models based on physical principles and differential equations. Authors in [21] had already discussed this question and identified challenges and fundamental parameters of the neural network. They used Lorenz ´95 (low-dimensional dynamical system introduced by Edward Lorenz in 1995) system for data assimilation to simulate climate phenomenon for air temperature prediction. This approach improved the forecast prediction based on a huge dataset of measured data from meteorological observations, but in fact, the proposed model completely substituted ECMWF for local prediction—which is not a primary task of weather forecast modelling research. Work [22] presented a case study of air temperature forecast for Germany with data from 2006 to 2017. For their model, the authors used horizontal resolution of 35 km and analysed the importance of meteorological station location (altitude, orthography). The proposed system reached good results, but the output data had a close relation to the specific meteorological station, total cloud cover parameter, wind at 850 hPa, and prediction period of only 48 h.

All the above-mentioned research articles and studies helped us to identify the key parameters that served for neural network development process.

## 3. Methodology

### 3.1. Data Collection

Evaluation of forecast accuracy delivered by numeric weather model is executed by the following methods [2]:

- Percentage of accuracy expressed as a ratio between the predicted and measured value;
- pivot table providing data regarding the number of cases when the phenomenon was predicted either correctly or falsely.

**Figure 3:**
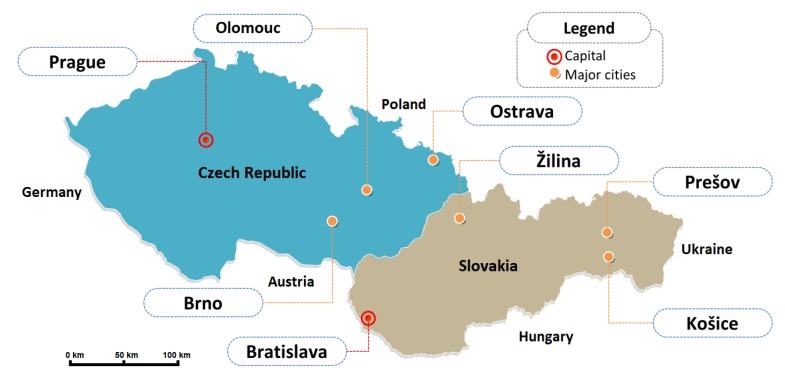
Location of selected cities.

For this purpose, we needed to collect the data from meteorological stations and compare them with model forecasts. Data collection took four months, the time period lasted from May to August 2019. We have chosen meteorological services of two nations—namely Slovak and Czech hydrometeorological institute—that offer forecasts of regional model ALADIN and global model ECMWF. Since ALADIN predicts meteorological parameters for three upcoming days, we have gathered forecast data from the ECMFW model for the next three days as well [1,3,23,24]. The data has been collected from meteorological stations of major cities in both countries (see Figure 3).

Selected cities overview can be seen in Table 1. [23,24]. Two cities, Olomouc (Czech Republic) and Prešov (Slovakia), were used for cross-validation, and they were not a part of the training and testing phase for neural network. Model verification was done by using the data collected during September 2019.

### 3.2. Analysis of Key Features Affecting the Urban Climate

Urban areas are inhabited by three-quarters of the European population. Soil profile in cities has a major impact on the local climate, especially in high-density housing estates. We can observe a gap between the predicted and measured air temperature in UHI. This gap is typically higher in comparison with rural areas because of the absence of green infrastructure. The published articles mentioned in the second section confirmed the importance of vegetation zones in cities. They also pointed at negative effects of the UHI on air quality caused by the UHI releasing air pollutants, which leads to average temperature growth [15,16,17,20]. 

The European Environment Agency (EEA) is a European Union agency whose main goal is to provide information on the environment and support the sustainability of natural resources. The Urban Atlas supported by EEA provides details about the urban land use in a form of high-resolution maps. These maps contain detailed information about the green area typology (city forests and gardens, parks and zoos, excluding public cemeteries that are used as public green zones in some cities) [25]. 

**Figure 4:**
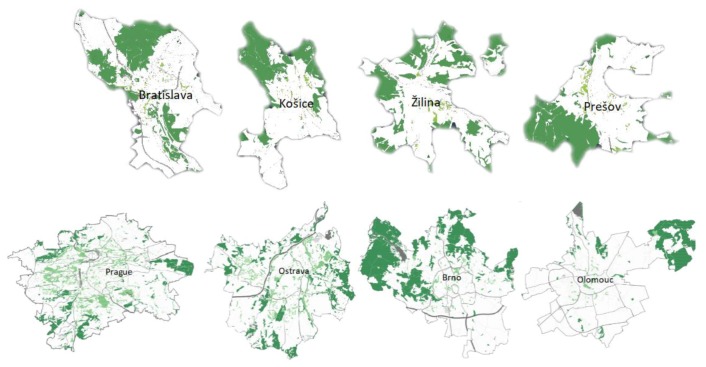
Green infrastructure of selected cities.

We have selected the ratio of green areas within cities as an input parameter that can help the air temperature forecast from global model to adjust to local conditions. Another important element of the local ecosystem influencing geophysical processes is water availability. Regional climate changes define new patterns of surface hydroclimate with local increment or reduction expected in precipitation and runoff. Vegetated and water areas (GI—green infrastructure) in cities produce a cooling effect thanks to evapotranspiration and shadowing, which improves the thermal comfort of urban residents and raises their resilience to heatwave events. GI areas are unsealed with high absorption of potential stormwater (trees, grass, or retention basin). Table 2 shows the percentage of green urban areas and water areas in our selected cities, and Figure 4 shows the visualization of GI in the mentioned cities [25].

In urban areas, the concentration of particulate matter ($PM_{2.5},\;PM_{10}$*)* particles, ozone ($O_{3}$), or Nitrogen dioxide ($NO_{2}$) are closely related to the emissions from traffic and factories concentrated in heavy industry. EEA has defined four stages of air quality index: Good, fair, moderate, and poor. This index accumulates data for the last 30 days and provides useful information on which air gases elements overcome air quality standards and may cause local differences between measured and forecast air temperature or precipitation [25,26].

### 3.3. Data Integration

This subsection describes the process of combining data from different sources into a single unified view. Integration begins with the ingestion process, and it includes steps based on mapping and transformation of cleaned dataset.

In order to design neural network capable of improving the next three-day forecast obtained from the global model (which corresponds with the forecast time period of regional models), we needed to define the air quality index (AQI) in the required form. At first, we constructed an AQI scale ranging from 1 to 5 (5—Good, 4—Moderate, 3—Unhealthy for Sensitive Groups, 2—Unhealthy, 1—Very Unhealthy), then we defined weighed air quality index (WAQI) average formula:

(1) $$\mathrm{WAQI}=\frac{\sum_{i=1}^{n}w_{i}x_{i}}{\sum_{i=1}^{n}w_{i}}$$

where:

$w_{i}$—weights expressed as percentage of selected air quality index level

$x_{i}$—value corresponding with AQI scale

For each selected city, WAQI value was computed based on the last-30-day pollution summary referring to the day prior to the first day of forecast. ECMWF delivers forecast updates two times per day, 0:00 UTC (coordinated universal time) and 12:00 UTC, for the next 10 days with 3 h prediction step. Since the main aimed contribution of this work was to make ECMWF model more accurate and to get closer to ALADIN regional model outputs, we collected data under the same circumstances (three-day period and forecast run at 00:00 UTC). We compared the predicted air temperature and precipitation with 3 h step (e.g. 3:00–6:00–9:00 pm, etc.), although ALADIN was offering the forecast with 1 h step. All the necessary parameters included in our dataset are presented in Table 3.

Except from ECMWF and ALADIN, we also included a forecast product distributed by the Norwegian Meteorological Institute—yr.no—into our comparison. This service is popular due to its free world forecast accessibility. Yr.no provides a high-resolution forecast for Nordic region by using NWP model Applications of Research to Operations at Mesoscale (AROME). Its forecast outputs are processed more precisely thanks to bilateral cooperation between Swedish and Norwegian national institutions system called Meteorological Cooperation on Operational Numerical Weather Prediction (MetCoOp). Outside this region, the ECMWF model with satellite images from the European Organisation for the Exploitation of Meteorological Satellites (EUMETSAT) is used. The forecast is typically slightly different from “raw” ECMWF data (and shows 1 h step prediction), therefore we wanted to analyse the accuracy of ECMWF and yr.no outputs.

### 3.4. Neural Network Modelling

The database of predicted and measured values for air temperature and overall daily precipitation is used for the neural network (NN) designing not only as input but also as a reference for supervised learning algorithm. This algorithm compares model outputs with the desired outputs, and according to the comparison it modifies the NN mapping function. 

**Figure 5:**
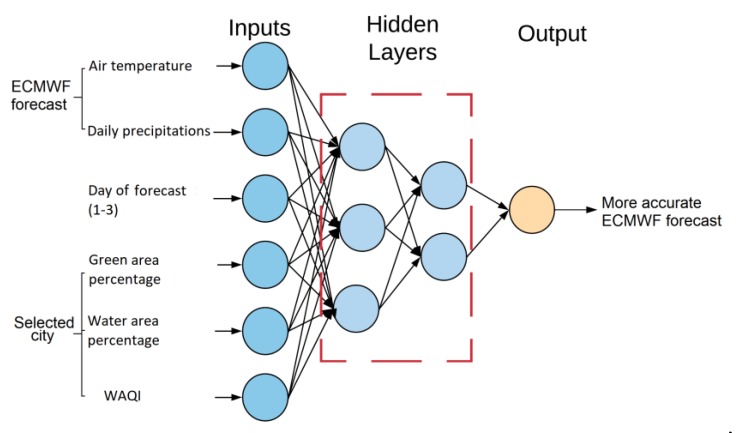
Neural network diagram.

The multi-layer perceptron (MLP) is a feedforward neural network that includes at least three layers (input, hidden, and output layer). Feedforward mechanism points to data transfer from the input to the output layer. The main contribution of neural networks lies in the iterative learning process in which the input data is sent to the network one at a time, and the weights associated with the input parameters are modified each time. Benefits of neural networks include their adaptability to noisy data or their versatility to classify (for model unknown) input data which have not been used for NN modelling. As it can be seen in Figure 5, database data serving as inputs for NN consist of air temperature and precipitations forecast, day of forecast (1–3), green and water area percentage of the selected city, and WAQI. As for outputs, air temperature and precipitation (with higher accuracy) would be calculated and compared with real data gained from the dataset.

On the other hand, the approximation given by the neural network will not give any insight into the form of computed approximation function. There is no simple link between the weights and the function being approximated. From a traditional statistics viewpoint, a neural network is a non-identifiable model. Given a dataset and network topology, there can be two neural networks with different weights and same result. As an example of "non-black box models", regression equation can be used. This conventional model gives a formula where the importance of each element is explicit. NN are very difficult to be interpreted and it is challenging to identify which input parameters are the most important and how they are related to the property being modelled. Therefore, we used datamining principles to identify key input elements that have impact on approximation procedure created by NN.

## 4. Results

At first, the basic statistical investigation was performed on the collected data. This investigation compares total precipitation and air temperature with the measured values obtained from weather stations. The accuracy (scale 0–1, 1 represents 100 % accuracy) of the numerical model is given by the following formula:

(2) $$Acc=\;\frac{Value_{predicted}}{Value_{measured}}$$

The aim of this method is to show which model predicts this local phenomenon with greater veracity. Calculated results are shown in Table 4 and Table 5.

The second method we have used is table of confusion. This method reports the number of false positives, false negatives, true positives, and true negatives. This allows more detailed analysis than relative accuracy of the forecast because if the data set is uneven, it can lead to wrong interpretation of measured data (e.g., when the numbers of observations in different classes differ greatly).

In our case, forecast outputs can be divided into four groups [2]:

- A: Intervention—number of cases when the phenomenon was predicted and really happened. Good forecast.
- B: Error—the phenomenon was not predicted and occurred. Wrong forecast.
- C: False—the phenomenon was predicted and did not arise. Wrong forecast.
- D: Correct—the phenomenon was not predicted and did not happen. Good forecast.

With respect to the Meteoalarm (alerting Europe system for extreme weather) web-based service, we set extreme values that represented investigated phenomenon incidences as follows [23,24]:

- hour air temperature: ≥ 30 °C
- amount of daily precipitation: ≥ 20 mm/24 h

These values represent the first warning stage (from 3) that means that the weather is potentially dangerous.

Outputs of this method are shown in Table 6. We selected two verification methods, namely probability of detection (POD) and false alarm ratio (FAR) [1]. Both can reach value ranging from 0–1. POD is defined as (higher value is better):

(3) $$POD=\;\frac{a}{a+b}$$

And FAR is described as (lower value is better):

(4) $$FAR=\;\frac{c}{a+c}$$

As it can be seen from Table 6, numerical model ALADIN gained the best results in extreme weather events forecast.

Neural networks can solve many recognition problems (images, emotions), create approximation function, substitute regression analysis, etc.

Determination of the number of layers and neurons per layer is decisive. The selected final topology reflects the best choice in terms of computing complexity and prediction veracity. In our case, testing on tens of topologies (with five to fifty neurons per layer and three to six layers) were performed. Each layer was a weighted sum of all the neurons (nodes)*j* from the previous layer, plus a bias (bias or constant is set to shift the result of activation function towards the positive or negative side) term [5]:

(5) $$Y=\;\underset{j}{\sum }w_{j}x_{j}+bias$$

The first layer represents inputs or features, the last one delivers output in a form of selected prediction parameter. The activation function of each node represents the activation potential of the node that influences whether the neuron will be fired or not. Nonlinear activation function allows to create a neural network as a function approximator. Our database consisted of 1188 collected samples. We analysed and cleared edge data with small number of occurrences, temperature range was set from 15–35 °C and daily precipitation forecast modelling started from 2 mm.

For training validation and testing phase, dataset was randomly divided into a percentage 80–10–10. We tested three training functions (activation function was set to default MATLAB tan-sigmoid), namely Levenberg–Marquardt optimization algorithm (requires more memory but less computing time), Bayesian regularization (suitable for small or noisy data but requires more time), and Scaled conjugate gradient (very quick, less memory consumption). Only the first mentioned training function reached R value better than 0.9 for air temperature and 0.8 for total daily precipitation. The training and validation were repeated 10–15 times with regard to overfitting for which several techniques were applied (e.g., gradient descent and cross-validation). The testing set (hour air temperature) achieved the highest correlation with Pearson’s Coefficient (around 0.915) and RMSE of 2.06 ($RMSE=\;\sqrt{MSE\left(\Theta \right)}$).

**Figure 6:**
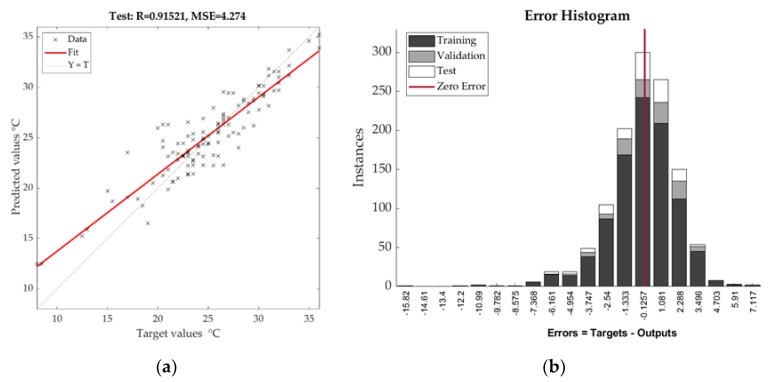
Correlation diagram (**a**) and error histogram (**b**) of NN (neural network) testing phase for air temperature.

**Figure 7:**
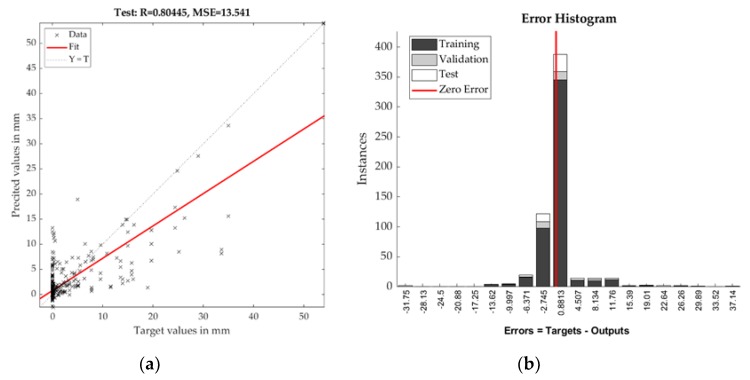
Correlation diagram (**a**) and Error histogram (**b**) of NN testing phase for daily summary of precipitation.

The testing set of daily precipitation reached R slightly over 0.8 with RMSE of 3.68. Model outputs are depicted by Figure 6 and Figure 7.

### Cross-Validation of the Proposed Enhancement ECMWF Forecast Model

Cross-validation is a model verification procedure used to assess how the model will generalize the data from the independent dataset. It is important to evaluate forecast accuracy using genuine forecasts. The accuracy of forecasts can only be determined by considering how well a model performs on new data that were not used when fitting the model. A forecast “error” is the difference between an observed value and its forecast. In this case, “error” does not mean a mistake, it means the unpredictable part of an observation.

For this purpose, we used two cities (one from both countries) and the weather data collected during September 2019 [23,24]. The results (in the form of percentage of accuracy) shown in Table 7 and Table 8 confirmed satisfactory accuracy level of our model and verified forecast improvement in comparison to standard ECMWF numerical model.

## 5. Discussion

Our model based on neural network reached better accuracy than the “classic” ECMWF model. We were able to identify important features for model inputs, which led to more precise production of forecast outputs. This work served as a proof of concept, and the results pointed out the practical potential of our main idea regarding the usage of neural network for weather forecast development. The proposed model based on neural network can compute and deliver enhanced ECMWF forecast output immediately, and thanks to elimination of the need for special supercomputing power it can potentially reduce operating costs of national weather services. Due to the climate change, this research field remains actual and the paper presents a novelty approach related to this issue.

Obtained results pointed to an interesting fact that global numerical forecast model ECMWF and its derivation yr.no can be a successful competitor to local models such as ALADIN. Limited area forecast models require high-performance computing which causes huge initial costs for every member country. This paper compares pros and cons of forecast numerical modelling.

We have identified a few limitations of numerical forecast modelling. In the case of overall precipitation forecast, “midnight forecast error” may occur sometimes. If a numerical model predicts precipitation starting e.g., at 11:00 pm and it really happens at 1:00 am of the next day, 2 h delay causes that precipitation amount to become part of the next weather station observation measurement. Another limitation directly related to our ECMWF model enhancement application is a small range of WAQI parameter. Although we tested eight cities in two countries, we did not obtain high variability of this parameter. Due to that, we intend to continue with data collecting especially in winter, when the pollution is typically higher than during the rest of the year due to weather inversion and heating period. We plan to carry on with research in this field and implement an extended version (based on the comments and ideas gained from real deploying) of the application suitable for portable devices.

## 6. Conclusions

This work proposes a forecast model based on machine learning and sensor data processing. The novelty of this approach is focused on forecast accuracy improvement of global numeric model ECMWF. The application can deliver outputs (in a form of more accurate ECMWF prediction) in real-time, and it is easy to use by both professionals and basic end-users. Several freely accessible applications use the ECMFW data and visualize it. Although local weather models typically release better forecasts, they require a high level of computer performance and the data are only available for each country separately. Our implemented classifier improves the ECMWF prediction accuracy for small and medium-size towns and brings better orthographic projection adaptability, which is the main advantage of limited area forecast models. This benefit can save costs for national meteorological stations and it improves visibility of forecast outputs to a wide spectrum of potential end-users.

## Figures and Tables

**Table 1:** Basic information about the selected cities [23,24].

City/Weather Station	Population (Thousands)	Area (Square km)	Altitude (Metres above Sea Level)	ALADIN Model Altitude	ECMWF Model Altitude
Prague/Prague-Karlov Brno/Brno-Žabovřesky Ostrava/Slezká Ostrava	1309 381 289	496 230 214	260 236 269	190 214 219	262 273 227
Bratislava/Brat. Mlynská Dolina	433	367	182	160	161
Žilina/Žilina mesto Košice/Košice mesto *Olomouc/Olomouc Holice*	81 239 101	80 243 100	365 203 210	386 242 225	509 327 230
*Prešov/Prešov vojsko*	89	70	307	291	386

**Table 2:** Green infrastructure and air quality index for selected cities [25,26].

City	Percentage of Green Urban Areas	Percentage of Urban Water Areas	Air Quality Index—30 Days Summary (Example: August 31)
Prague Brno Ostrava	18.77 30.63 20.16	1.5 1.4 2.3	60%—Good, 39%—Moderate, 1%—Unhealthy, 53%—Good, 46%—Moderate, 1%—Unhealthy 56%—Good, 42%—Moderate, 1%—Unhealthy for Sensitive Groups, 1%—Unhealthy
Bratislava	11.05	4.5	57%—Good, 43%—Moderate
Žilina Košice *Olomouc*	33.25 16.15 13.54	4.2 0.6 1.2	57%—Good, 43%—Moderate 50%—Good, 49%—Moderate, 1%—Unhealthy for Sensitive Groups 77%—Good, 22%—Moderate, 1%—Unhealthy for Sensitive Groups
*Prešov*	37.51	0.4	43%—Good, 57%—Moderate

**Table 3:** List of dataset parameters.

Type of Parameter	Description
Forecast meteorological variables: Forecast models (meteograms) Measuring system (forecast verification) Forecast period Forecast run Air pollution impact	Air temperature (3 h step), precipitation (24 h summary) ECMWF, ALADIN, yr.no Weather stations in selected cities (inner-city location) 1^st^, 2^nd^, or 3^rd^ day 00:00 UTC WAQI
Green infrastructure	Vegetation and water areas ratio

**Table 4:** Models forecast comparison for air temperature.

Numerical Model	Relative Accuracy of the Forecast
ALADIN (SK+ CZ) ECMWF Yr.no	0.9921 0.9857 0.9956

**Table 5:** Models forecast comparison for daily precipitation.

Numerical Model	Relative Accuracy of the Forecast
ALADIN (SK+ CZ) ECMWF Yr.no	0.7292 0.824 0.7824

**Table 6:** Accuracy of extreme weather forecast. The best results are bolded.

Model	ALADIN		ECMWF		YR.NO	
Variable	T/°C	mm/24 h	T/°C	mm/24 h	T/°C	mm/24 h
	Number of Occurrences					
a	**120**	**6**	92	2	98	2
b	**28**	**11**	51	15	36	15
c	27	9	**14**	**0**	25	4
d	1013	568	**1031**	**577**	1029	573
POD	**0.811**	**0.353**	0.643	0.118	0.731	0.118
FAR	0.184	0.6	**0.132**	**0**	0.203	0.667

**Table 7:** Cross-validation results for air temperature forecast.

Numerical Model	Relative Accuracy of Forecast
ALADIN (SK+CZ)	0.9637
ECMWF	0.953
Yr.no	0.9876
**Our Model**	**0.9678**

**Table 8:** Cross-validation results for daily precipitation forecast.

Numerical Model	Relative Accuracy of Forecast
ALADIN (SK+CZ)	0.783
ECMWF	0.6916
Yr.no	0.8152
**Our Model**	**0.7689**
